# Efficacy and safety of probiotic supplementation in PsA—a randomized controlled trial

**DOI:** 10.1093/rheumatology/keag547

**Published:** 2026-10-05

**Authors:** Philipp Bosch, Angelika Lackner, Barbara Dreo, Johannes Fessler, Florentine Moazedi-Fürst, Rusmir Husic, Joana Séneca, Andreas Haidmayer, Vanessa Stadlbauer, Anirudh Subramanian Muralikrishnan, Jens Thiel, Martin Stradner

**Affiliations:** Department of Rheumatology and Immunology, Medical University of Graz, Graz, Austria; Department of Rheumatology and Immunology, Medical University of Graz, Graz, Austria; Department of Rheumatology and Immunology, Medical University of Graz, Graz, Austria; Division of Immunology, Otto Loewi research Center, Medical University of Graz, Graz, Austria; Department of Rheumatology and Immunology, Medical University of Graz, Graz, Austria; Department of Rheumatology and Immunology, Medical University of Graz, Graz, Austria; Joint Microbiome Facility, University of Vienna, Vienna, Austria; Department of Laboratory Medicine, Medical University of Vienna, Vienna, Austria; Department of Microbiology and Ecosystem Science, Centre for Microbiology and Environmental Systems Science, University of Vienna, Vienna, Austria; Department of Rheumatology and Immunology, Medical University of Graz, Graz, Austria; Division of Gastroenterology and Hepatology, Department of Internal Medicine, Medical University of Graz, Graz, Austria; Center for Biomarker Research in Medicine (CBmed), Graz, Austria; Department of Rheumatology and Immunology, Medical University of Graz, Graz, Austria; Department of Physiology & Pathophysiology, Otto Loewi Research Center for Vascular Biology, Immunology, and Inflammation, Medical University of Graz, Graz, Austria; Department of Rheumatology and Immunology, Medical University of Graz, Graz, Austria; Department of Rheumatology and Clinical Immunology, Medical Center-University of Freiburg, Faculty of Medicine, Freiburg, Germany; Department of Rheumatology and Immunology, Medical University of Graz, Graz, Austria

**Keywords:** PsA, probiotics, microbiome, randomized controlled trials, disease activity

## Abstract

**Objectives:**

To evaluate the clinical efficacy and safety of a multispecies probiotic in patients with PsA in moderate disease activity.

**Methods:**

In this single-centre, randomized, double-blind, placebo-controlled trial (RCT), 66 PsA patients with moderate disease activity (Psoriatic Arthritis Disease Activity Score [PASDAS] > 3.2 to <5.4) on stable immunomodulatory therapy were randomized 1:1 to receive either a multispecies (*Lactobacillus*, *Bifidobacterium*) probiotic or placebo for 12 weeks. The primary outcome was treatment response at week 12, defined as achieving low disease activity or remission (PASDAS ≤3.2) without treatment change. Secondary outcomes included gut permeability markers, immune cell and microbiome composition. Analyses were conducted per a modified intention-to-treat (only complete cases for primary outcome), with sensitivity analyses using multiple imputation, per-protocol datasets and qPCR-based detection of the probiotic strains in stool (adherence).

**Results:**

Fifty-six participants were included in the main analysis. Thirteen (43%) participants in the probiotic group and 17 (65%) in the placebo group achieved the primary outcome (*P* = 0.167). The corresponding odds ratio was 0.4 (95% CI 0.13–1.18). Patient-reported pain and global assessment were the main PASDAS components improving over time, with no between-group differences. No differences were observed in secondary clinical outcomes, gut permeability markers (zonulin, calprotectin, alpha-1-antitrypsin), immune cell profiles or stool microbiome composition between the groups at week 12. No serious adverse events related to the study product were reported.

**Conclusion:**

In this first RCT investigating probiotic supplementation in PsA, probiotic treatment did not show superiority over placebo in achieving treatment response, or altering gut microbiome, or permeability.

*Rheumatology* key messages

- The tested multispecies probiotic is not superior to placebo in improving PsA clinical disease activity after 12 weeks.
- Treatment over 12 weeks does not improve secondary clinical outcomes, gut permeability markers, immune cell profiles or stool microbiome composition compared to placebo.
- No new safety signals were observed under the tested probiotic treatment.

## Introduction

PsA is a chronic inflammatory musculoskeletal disease characterized by peripheral arthritis, axial disease, enthesitis and dactylitis. Subclinical gut inflammation is present in ∼20% of patients [1]. Recent Mendelian randomization analyses suggest that gut inflammation is a causal factor in the pathogenesis of PsA and psoriasis, supporting the concept of the gut–joint axis as a key pathogenic driver of disease [2, 3]. This is further supported by the co-occurrence of arthritis and gut inflammation in the TNF-transgenic and SKG mouse models of SpA [4, 5]. Similar results have also been described in psoriasis.

Disease onset and activity in PsA are hypothesized to be associated with bacterial dysbiosis—an alteration or disruption of the normal microbiome—with reduced abundances of *Ruminococcus* and *Akkermansia* species in the ileum of patients with PsA compared with patients with psoriasis only and healthy controls [6, 7].

Probiotics, per definition, are live microorganisms that, when administered in adequate amounts, confer a health benefit on the host, and a recent meta-analysis suggests a benefit in inducing remission in ulcerative colitis [8, 9]. *Bifidobacterium* and *Lactobacillus* species are the most commonly used strains due to their favourable safety profile and global interest is high, with the probiotics market valued at USD 87.70 billion in 2023 and projected to reach USD 220.14 billion by 2030. Although their use has steadily increased among patients with chronic inflammatory conditions, including PsA and RA over the last decade, clear evidence of clinical benefit is lacking [10, 11].

We conducted an open-label pilot study demonstrating improvements in clinical outcomes and gut permeability markers following probiotic supplementation in patients with PsA [12]. The probiotic supplement was chosen pragmatically, as a widely used over-the-counter multispecies probiotic frequently taken by our patients, rather than for hypothesized strain-specific mechanisms.

The aim of this study was to assess clinical efficacy, safety and secondary microbiome and gut parameters in a randomized controlled trial (RCT) setting.

## Methods

### Study design and participants

MEDIPSA (Modulation of Enteral Dysbiosis on Disease Activity in Patients with Psoriatic Arthritis) was a single-centre, parallel-group, placebo-controlled, investigator initiated, superiority randomized trial with a 1:1 allocation ratio.

Patients were recruited from the outpatient clinic of the Department of Rheumatology and Immunology at the Medical University of Graz—a tertiary care centre in Austria. Eligible participants were 18–89 years old, diagnosed with PsA, fulfilling the ClASsification criteria for Psoriatic ARthritis (CASPAR) and had moderate disease activity (MoDA, Psoriatic Arthritis Disease Activity Score (PASDAS) > 3.2 to <5.4) with stable immunomodulatory therapy for at least 12 weeks prior to baseline (i.e. no changes in biological or synthetic disease modifying drugs (bDMARD or sDMARD), or glucocorticoid treatment) [13, 14]. Exclusion criteria included bariatric surgery, use of probiotics within 24 weeks and antibiotic use within 4 weeks prior to inclusion, inflammatory bowel disease, recent acute myocardial infarction, stroke or decompensated heart failure (within 12 weeks), known malignancy and pregnancy.

Eligible patients provided a written informed consent prior to study inclusion. The study conformed to the principles of the Declaration of Helsinki, its protocol was registered to ClinicalTrials.gov prior to study initiation (NCT04588623, date of registration: 08.10.2020) and received ethical approval from the Medical University of Graz (32–347 ex 19/20).

### Randomization and masking

After written informed consent for trial participation was obtained, eligible patients were randomized to receive either an over-the-counter multispecies probiotic (see Supplementary Data S1 for details on the probiotic and placebo composition) or a matching placebo, taken once daily for 12 weeks, with the option to continue probiotic treatment for an additional 12 weeks in an open-label phase.

Block randomization using fixed block sizes was performed centrally in a 1:1 ratio with use of a computer random-sequence generator, by a person not otherwise involved in the study [15]. Sequentially numbered, opaque, sealed envelopes containing a kit number for each participant were used. Probiotic and placebo were packed identically, randomized, labelled with a kit number and had the same appearance, taste and smell. The blinded study personnel dispensed the study medication based on the kit number obtained by opening the envelopes in sequence after inclusion of the participant into the study. The participants, caregivers and all investigators, including data collectors and outcome assessors, were blinded until completion of the primary data analysis.

Patients were instructed to dissolve the study medication in a glass of water once daily, wait 1 min before drinking and take it on an empty stomach, either in the morning before breakfast or in the evening.

### Procedures

Study visits occurred at baseline, 12 and 24 weeks, with telephone follow-ups at weeks 6 and 18 to monitor adherence, record adverse events and address any concerns. At each in-person visit, clinical symptoms, changes in medical therapy, adverse events, clinical and patient-reported outcomes were documented. Additionally, blood was drawn for routine parameters and for the analysis of T cell distribution, phagocytosis testing and a stool sample was collected for microbiome analysis and gut permeability markers. The microbiome analysis, DNA extraction and sequencing was performed at the Joint Microbiome Facility at the University of Vienna.

### Outcomes

The primary outcome was treatment response, defined as the change from moderate disease activity at baseline, assessed by baseline PASDAS [16], to low disease activity or remission (LDA/REM, PASDAS ≤3.2) at week 12 without a disease flare during this period resulting in a treatment change.

Demographic information, including dietary intake was collected, with ethnicity self-reported by the participants using an open-ended question. Clinical outcomes included Tender Joint Count (TJC, 68 joints), Swollen Joint Count (SJC, 66 joints), Leeds Enthesitis Index (LEI) [17], Dactylitis Score (number of affected fingers and toes) [18], Psoriasis Area and Severity Index (PASI) [19], Patient and Evaluator Global Assessments of disease activity (PGA, EGA) and patient-reported pain (Pt-pain), all three on a 0–10 scale (10 representing worst disease activity/pain), the Health Assessment Questionnaire (HAQ) [20], Dermatology Life Quality Index (DLQI) [21], the Psoriatic Arthritis Impact of Disease 12-item questionnaire (PSAID) [22], the Physical Component Summary of the Short Form-36 Health survey (SF-36 PCS) [23], the Functional Assessment of Chronic Illness Therapy for Fatigue (FACIT Fatigue) [24], the Hospital Anxiety and Depression Scale (HADS) [25], quantitative PASDAS values, disease activity in psoriatic arthritis (DAPSA) values [26] and minimal disease activity (MDA) criteria [27].

Prespecified secondary endpoints included changes from baseline to weeks 12 and 24 in gut permeability markers (zonulin, calprotectin, alpha-1 antitrypsin), T cell distribution and neutrophil function (phagocytosis and oxidative burst), as well as changes in the composition of the gut microbiome (for details see Supplementary Data S2 and S3).

For zonulin, calprotectin and alpha-1 antitrypsin data are shown quantitatively and also in terms of above or below the normal reference ranges (zonulin: <55 ng/ml; calprotectin: <50 mg/l; alpha-1 antitrypsin: <27.5 mg/dl) [28–30]. Samples were measured in duplicates by ELISA at baseline and week 12. Only values with ≤20% deviation between duplicates were retained.

Adverse events were recorded according to the Medical Dictionary for Regulatory Activities (MedDRA) Version 28.1 and causality to the study medication is depicted according to World Health Organization-Uppsala Monitoring Centre (WHO-UMC) scale [31, 32].

All outcomes were collected at baseline, week 12 and week 24.

### Statistical analysis

A sample size of 56 participants was estimated to provide 80% power to detect an effect size of 0.4 (60% treatment response in the probiotic and 20% in the placebo arm) with an alpha of 0.05 using the continuity corrected *Z*-test with pooled variances. The effect size was estimated using data from a previous study [12]. An additional 30% of participants was recruited to account for lost to follow-up.

All randomized participants were analysed by assigned group, following exclusion of five ineligible participants, with the primary analysis restricted to complete cases for the primary outcome and missing data addressed through multiple imputation in the sensitivity analyses. The primary analysis compared treatment response at week 12 between the groups according to a modified intention-to-treat (complete case for primary outcome) analysis using Pearson’s *χ*^2^ test with Yates’ continuity correction. Secondary outcomes were analysed in an exploratory manner using effect estimates with 95% CIs, without adjustment for multiplicity. Univariable and multivariable logistic and linear regression models—including relevant covariates that differed between groups at baseline (i.e. disease duration in months, HAQ score, FACIT fatigue score and HADS-D score) and treatment variables as prespecified in the protocol (NSAIDs treatment [yes/no] and b/sDMARD treatment [yes/no])—were used to obtain odds ratios, effect sizes and 95% CIs for treatment response and quantitative PASDAS values at week 12 according to the modified intention-to-treat complete-case analysis. The same models were applied to the secondary outcomes DAPSA, MDA, SJC and CRP at week 12. Sensitivity analyses for treatment response and PASDAS values included a per-protocol analysis, an intention-to-treat analysis using multiple imputations for missing data and an analysis restricted to participants in the probiotic group who had at least one detectable probiotic strain in their stool at week 12 by qPCR (for details see Supplementary Data S3). Participants already on immunomodulatory treatment at enrolment were expected to remain on a stable regimen. Any change in the DMARD treatment before week 12 for reasons other than a flare led to exclusion from the per-protocol analysis. Multiple imputation was used to create and analyse five multiply imputed datasets as recommended [33]. Incomplete values were imputed under fully conditional specification using the default settings of the mice 3.18.0 and miceadds 3.17–44 packages assuming missingness at random [33]. Missing parameters (i.e. treatment response or PASDAS values at week 12 [missing in four cases that withdrew from the study] and NSAID treatment [missing in eleven cases]) were estimated in each imputed dataset separately, and combined using Rubin’s rules. Patients who were erroneously included in the study and did not fulfil the in/exclusion criteria, were retrospectively excluded from all analyses, except for the reporting of potential adverse events.

For the microbiome analysis, alpha diversity metrics such as the Chao1 richness estimator and the Shannon diversity index were assessed for each group. Differences in microbial community structure (i.e. beta diversity analysis) between the groups were evaluated using a PcoA based on Aitchison distances (for details see Supplementary Data S3). Relative abundance of genera, which were either found in the probiotic (*Bifidobacterium*, *Lactobacillus*) or were known to be reduced in PsA compared with healthy controls (*Akkermansia*, *Ruminococcus*), were visualized using bar plots [6].

All other analyses are depicted in a descriptive manner using mean (standard deviation), median (range) or number (%).

All analyses were performed using R 4.5.0 [34].

## Results

### Clinical outcomes

Patients were enrolled between 25 November 2020 and 9 September 2022. In the open-label phase, the final assessments were completed on 14 March 2023. A total of 105 individuals were screened, and 66 were randomized (see Fig. 1 and Supplementary Fig. S1). One participant in the intervention arm and four in the placebo arm were retrospectively excluded from all analyses due to incorrect inclusion based on disease activity outside the eligibility range. Four participants withdrew from the study before week 12: one in the probiotic group due to a change in treating rheumatologist, and three in the placebo group, two following infections (borreliosis and upper respiratory tract infection) and one after a diarrhoea episode. The primary outcome analysis included 30 participants in the probiotic group and 26 in the placebo group. Twenty-six and 23 participants completed the optional open-label phase of the study until week 24 in the probiotic and placebo group, respectively.

**Figure 1:**
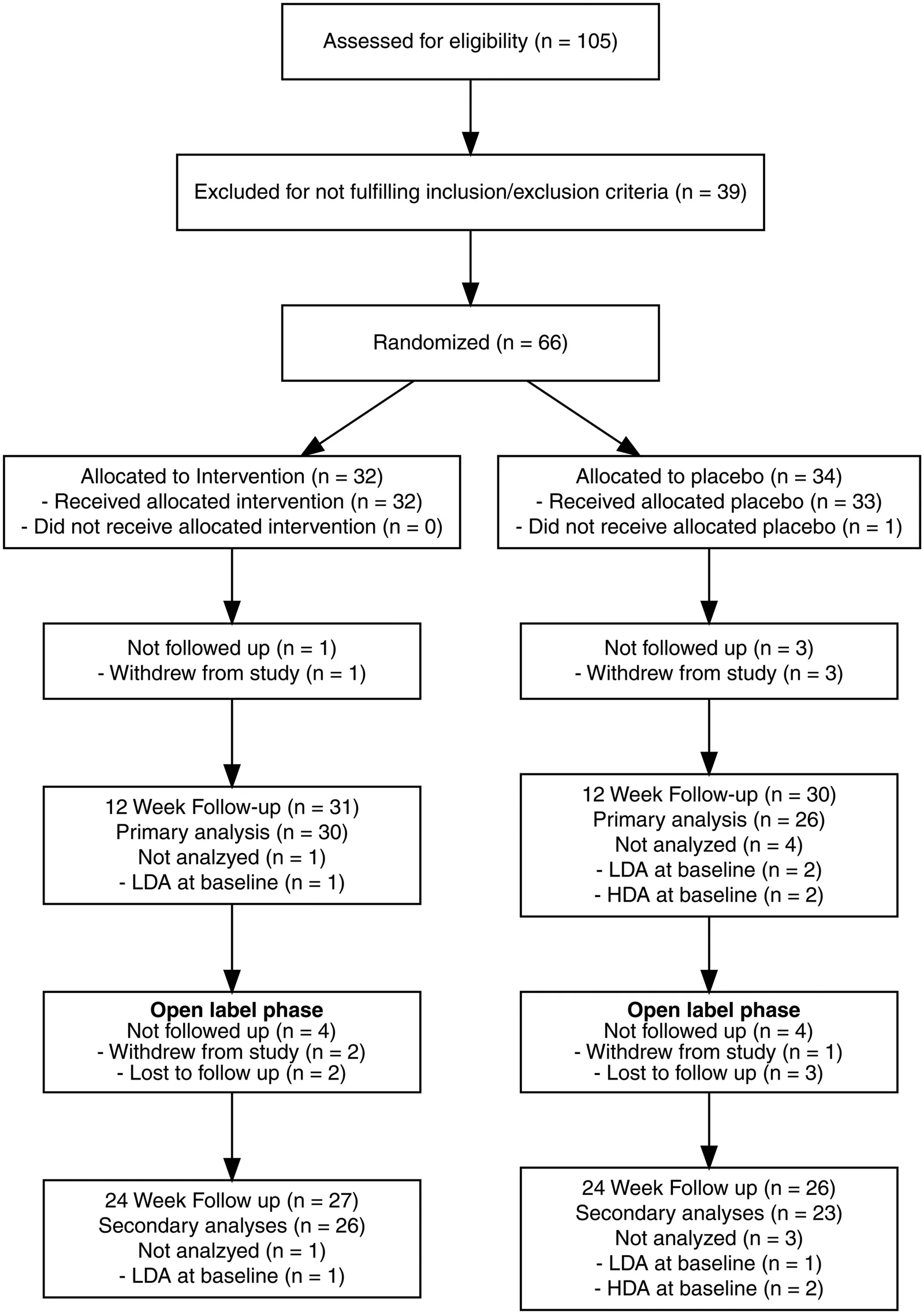
Flowchart according to the modified intention-to-treat complete-case analysis. HDA, high disease activity; LDA, low disease activity

Participants in the probiotic group were older, had a longer median disease duration, lower FACIT fatigue scores, higher HAQ and HADS scores and more frequently used bDMARDs and NSAIDs compared with those in the placebo group. Only two participants (8%), both in the placebo group, were vegetarian and all others were omnivorous. Dietary intake was otherwise comparable, aside from a slightly higher fish and lower sweet-dessert consumption in the probiotic group. All participants identified as White (see Table 1 and Supplementary Tables S1 and S2).

**Table 1:** Baseline characteristics.

Characteristic	Probiotic *N* = 30	Placebo *N* = 26	SMD
Age, years	53 (11)	48 (14)	0.39
Female subjects	19 (63%)	16 (62%)	0.04
BMI, kg/m²	28.0 (6.0)	27.6 (4.4)	0.08
Current smoker	7 (23%)	1 (4.0%)	0.59
Consumes alcohol regularly	19 (63%)	18 (72%)	−0.19
Vegetarian	0 (0%)	2 (7.7%)	−0.41
Disease duration, months	71 (0–384)	23 (0–421)	0.10
PASDAS	3.9 (3.2–5.2)	3.8 (3.3–4.9)	0.15
DAPSA	13.3 (8.1–25.5)	11.4 (8.3–22.0)	0.55
MDA	3 (13%)	4 (19%)	−0.18
Tender joint count	3.0 (1.0–14.0)	2.0 (0.0–11.0)	0.57
Swollen joint count	1.0 (0.0–5.0)	1.0 (0.0–5.0)	−0.11
Pain visual analogue scale	38 (10–80)	40 (30–50)	0.17
Patient global assessment	30 (10–80)	40 (30–60)	0.15
Evaluator global assessment	20 (0–40)	23 (20–40)	−0.28
Leeds Enthesitis Index	0 (0–2)	0 (0–4)	−0.02
Dactylitis count	0 (0–0)	0 (0–1)	−0.28
CRP, mg/l	2 (1–48)	2 (0–10)	0.49
ESR, mm/h	9 (2–27)	7 (1–31)	0.29
SF-36 PCS score	39 (20–47)	40 (21–55)	−0.42
PSAID-12 score	2.9 (0.6–7.4)	2.5 (0.3–4.8)	0.46
HADS-D depression score	6.5 (0.0–20.0)	4.0 (0.0–14.0)	0.59
HADS-D anxiety score	8.0 (0.0–20.0)	7.0 (0.0–13.0)	0.55
PASI score	0.0 (0.0–6.5)	0.0 (0.0–6.0)	0.13
FACIT fatigue score	30 (8–51)	39 (13–52)	−0.58
HAQ score	0.94 (0.00–1.88)	0.25 (0.00–1.25)	1.2
Biologic DMARD			0.59
None	12 (40%)	15 (58%)	
TNFi	9 (30%)	6 (23%)	
IL17i	7 (23%)	4 (15%)	
IL23i	2 (6.7%)	0 (0%)	
IL12 + 23i	0 (0%)	1 (3.8%)	
Conventional DMARD			0.59
None	21 (70%)	18 (69%)	
MTX	8 (27%)	5 (19%)	
LFN	0 (0%)	3 (12%)	
Targeted synthetic DMARD			0.13
None	25 (83%)	22 (85%)	
JAKi	2 (6.7%)	1 (3.8%)	
Apremilast	3 (10%)	3 (12%)	
Current NSAID intake	16 (59%)	7 (35%)	0.50

Mean (S.D.), median (min to max) or *n* (%).

Missing values: BMI was available for 51 (91%) of patients; ESR for 55 (98%) of patients; current smoker status for 55 (98%) of patients; regular alcohol consumption for 55 (98%) of patients; vegetarian status for 54 (96%) of patients; PSAID-12 score for 55 (98%) of patients; PASI score for 48 (86%) of patients; HAQ score for 53 (95%) of patients; MDA for 45 (80%) of patients and current NSAID intake 47 (84%) of patients.

This includes 56 patients according to the modified intention-to-treat complete-case analysis.

DAPSA: disease activity in psoriatic arthritis; FACIT: Functional Assessment of Chronic Illness Therapy; HAQ: Health Assessment Questionnaire; HADS-D: Hospital Anxiety and Depression Scale—Depression; IL12 + 23i: IL 12 and 23 inhibitor; IL17i: IL 17 inhibitor; IL23i: IL 23 inhibitor; JAKi: Janus Kinase inhibitor; LFN: leflunomide; MDA: minimal disease activity; PASI: Psoriasis Area and Severity Index; PASDAS: Psoriatic Arthritis Disease Activity Score; PSAID-12: Psoriatic Arthritis Impact of Disease—12 items; SF-36 PCS: Short Form 36 Physical Component Summary; SMD: standardized mean difference; TNFi: tumour necrosis factor inhibitor.

The primary outcome was achieved by 13 (43%) participants in the probiotic group and 17 (65%) in the placebo group (*P* = 0.167). PASDAS values were numerically lower at weeks 12 and 24 in the placebo group (see Fig. 2). Regression model results are presented in Table 2. In the univariable analysis with PASDAS as the dependent variable, the placebo group showed lower scores than the probiotic group, though the difference was not statistically significant after adjustment for relevant covariates (i.e. disease duration, NSAID use, DMARD treatment, HAQ, FACIT fatigue and HADS-D Scores). Sensitivity analyses yielded similar findings (see Supplementary Table S3 and S4). Exploratory analyses using DAPSA, MDA, swollen joint count and CRP at week 12 as outcome variables instead of PASDAS showed no clear benefit of probiotic treatment, consistent with the primary outcome (see Supplementary Table S3).

**Figure 2:**
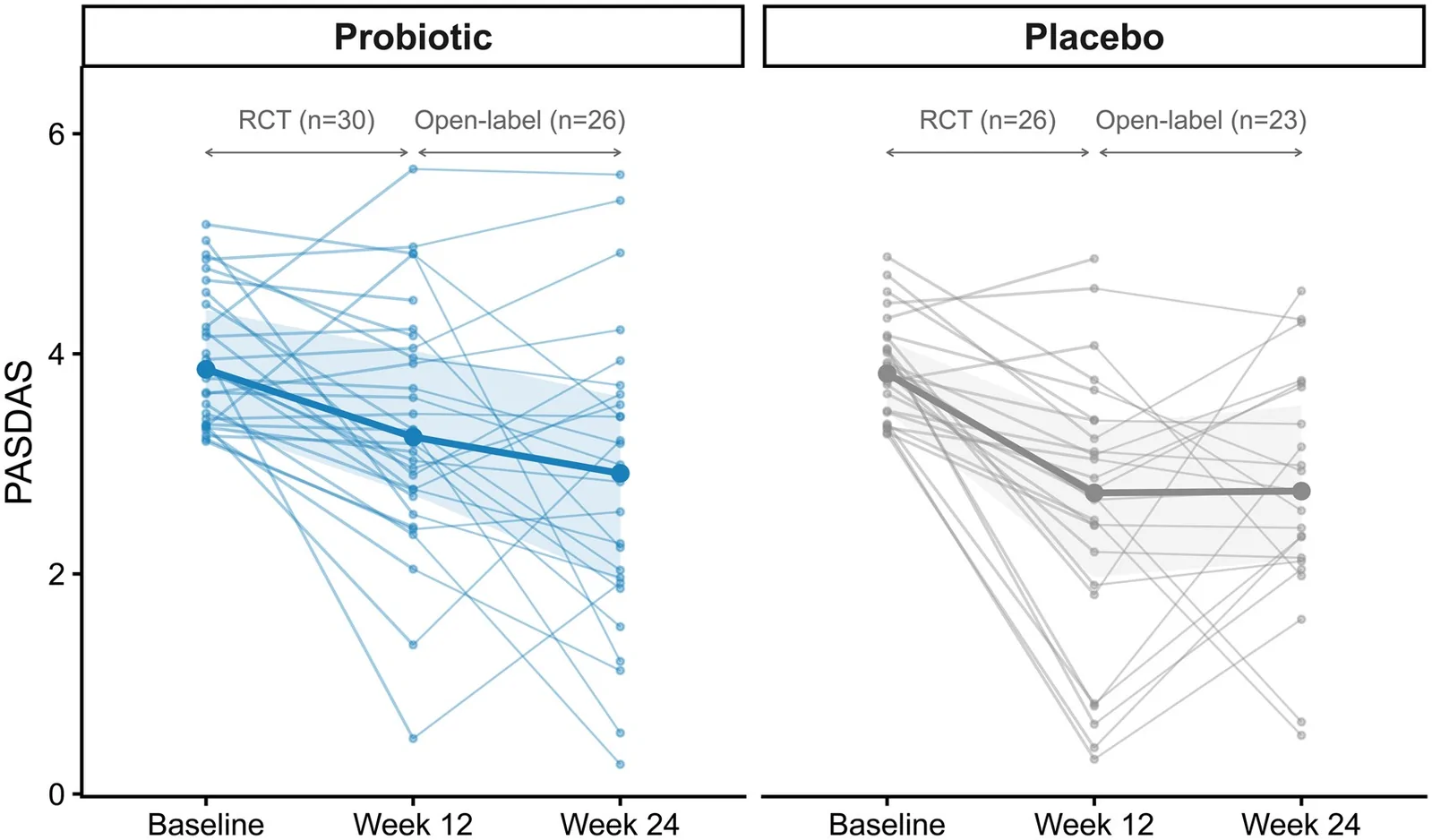
Individual participant PASDAS trajectories at baseline, week 12 and week 24, according to modified intention-to-treat complete cases. Thin lines show individual participants, the bold line shows the group median, with the shaded band representing the interquartile range. PASDAS, Psoriatic Arthritis Disease Activity Score

**Table 2:** Treatment response and PASDAS at week 12.

Outcome	*N* Probiotic	*N* Placebo	OR (95% CI)	Effect size (95% CI)
Treatment response^a^	30	26	0.4 (0.13–1.18)	NA
Treatment response^b^	25	19	0.79 (0.16–3.94)	NA
PASDAS^a^	30	26	NA	0.73 (0.1–1.35)
PASDAS^b^	25	19	NA	0.13 (−0.8 to 1.05)

Effect sizes and odds ratios refer to the difference between the probiotic and placebo groups. Treatment response: OR <1 indicates lower odds of response in the probiotic group compared with placebo. PASDAS: positive effect sizes indicate higher scores in the probiotic group compared with placebo.

a Univariable model for group assignment (probiotic or placebo) in the modified intention-to-treat complete-case analysis.

b Multivariable model for group assignment adjusting for DMARD treatment, NSAID treatment, disease duration, Health Assessment Questionnaire (HAQ) score, Functional Assessment of Chronic Illness Therapy (FACIT) fatigue score and Hospital Anxiety and Depression Scale—Depression (HADS-D) in the modified intention-to-treat complete-case analysis.

NA, not applicable; OR, odds ratio; PASDAS, Psoriatic Arthritis Disease Activity Score.

Among PASDAS components, PGA, Pt-pain and SF-36 PCS displayed a smaller numeric median improvement in the probiotic group compared with placebo, while other components showed similar changes between the groups. No secondary clinical outcomes differed between the groups from baseline to week 12 (see Table 3 and Supplementary Table S5). No relevant changes in clinical outcomes were observed from week 12 to week 24 in participants receiving probiotic treatment after starting the open-label phase (see Supplementary Table S6).

**Table 3:** Clinical outcomes at week 12.

	Probiotic		Placebo	
Outcome	*N*	Median (min–max)	*N*	Median (min–max)
PASDAS	30	3.2 (0.5–5.7)	26	2.7 (0.3–4.9)
Tender joint count	30	2 (0–13)	26	1 (0–11)
Swollen joint count	30	0 (0–7)	26	0 (0–4)
Pain visual analogue scale	30	30 (10–90)	26	20 (0–60)
Patient global assessment	30	29.5 (0–90)	26	20 (0–50)
Evaluator global assessment	30	19 (0–80)	26	10 (0–50)
Leeds Enthesitis Index	30	0 (0–3)	26	0 (0–0)
Dactylitis count	30	0 (0–0)	26	0 (0–0)
CRP	30	1.8 (0–24.3)	26	1.9 (0.4–14.9)
ESR	29	9 (2–19)	25	7 (2–23)
SF-36 PCS score	30	37.7 (22–53.1)	26	45.6 (24.8–57.6)
PSAID-12 score	27	2.8 (0.5–7.9)	26	1.3 (0–4.5)
HADS-D depression score	30	5 (1–19)	26	3 (0–11)
HADS-D anxiety score	30	7 (0–18)	26	5 (0–13)
PASI score	26	0 (0–3.8)	22	0 (0–2.7)
FACIT fatigue score	30	36 (4–50)	26	43.2 (17–52)
HAQ score	30	0.8 (0–2.8)	25	0.4 (0–1.5)

*N* represents the number of patients with available data per group for each outcome, according to the modified intention-to-treat complete-case analysis.

FACIT, Functional Assessment of Chronic Illness Therapy; HAQ, Health Assessment Questionnaire; HADS-D, Hospital Anxiety and Depression Scale; PASI, Psoriasis Area and Severity Index; PASDAS, Psoriatic Arthritis Disease Activity Score; PSAID-12, Psoriatic Arthritis Impact of Disease—12 items; SF-36 PCS, Short Form 36 Physical Component Summary.

### Gut permeability markers

Baseline zonulin levels exceeded the normal range in 54% of the probiotic group and 56% of the placebo group. Elevated calprotectin and alpha-1 antitrypsin were observed in 30% and 47% of participants in the probiotic group, and 33% and 33% of participants in the placebo group, respectively at baseline. At the 12-week follow-up, all markers remained similar, with slightly lower zonulin values in both the probiotic and placebo groups, and no differences between the groups (see Fig. 3 and Supplementary Table S7). No changes were observed in participants who received placebo followed by probiotics from week 12–24 (see Supplementary Table S8).

**Figure 3:**
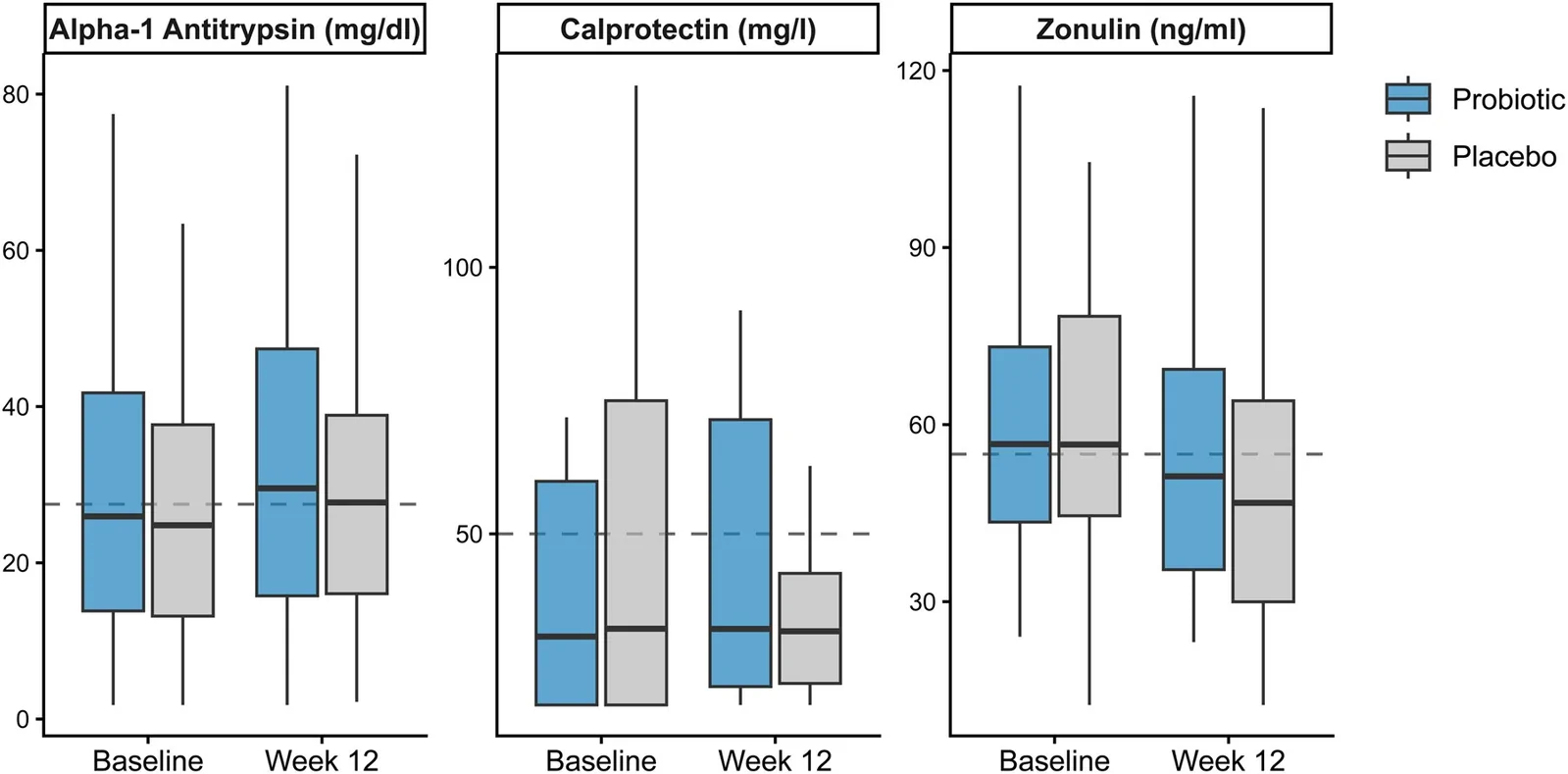
Gut permeability makers at baseline and week 12. The dashed line represents the normal reference range for each marker (higher values are considered abnormal). Normal reference ranges: zonulin: <55 ng/ml; calprotectin: <50 mg/l; alpha-1 antitrypsin: <27.5 mg/dl. Samples were measured in duplicates by ELISA at baseline and week 12. Only values with ≤20% deviation between duplicates were retained. Number of patients according to modified intention-to-treat complete-case analysis minus patients with duplicate deviation >20% (alpha-1 antitrypsin at BSL: 30 probiotic, 24 placebo; alpha-1 antitrypsin at W12: 30 probiotic, 25 placebo; calprotectin at BSL: 30 probiotic, 24 placebo; calprotectin at W12: 29 probiotic, 25 placebo; zonulin at BSL: 28 probiotic, 25 placebo; zonulin at W12: 29 probiotic, 25 placebo)

### Microbiome and immune cell analyses

No significant differences in microbial community richness or diversity were detected between the probiotic and placebo groups neither at baseline nor at week 12 (see Supplementary Fig. S2). Similarly, microbial community structure did not differ significantly between the groups (PERMANOVA, *P* > 0.05) (see Supplementary Fig. S3). Relative abundance of *Bifidobacterium*, *Lactobacillus*, *Akkermansia* and *Ruminococcus* was similar between both the groups at baseline and week 12 (see Supplementary Fig. S4).

In the probiotic group, stool DNA for qPCR was available for 23/30 participants at week 12 and at least one of the probiotic strains was detectable in 19/23. None of the probiotic strains were detected in stool samples from the placebo group at week 12. Running the analysis limited to probiotic-group participants with at least one detectable probiotic strain in stool yielded results consistent with the primary analysis for treatment response and quantitative PASDAS values.

T-cell subset composition including disease-relevant Th1, Th2, Th17 and Treg cells as well as phagocytosis tests were all similar between the probiotic and placebo group at baseline and no relevant changes were observed from baseline to week 12 in all participants and between week 12 and week 24 in participants in the placebo group receiving the probiotic in the open-label phase (see Supplementary Tables S9–S12).

### Adverse events

Adverse events were evenly distributed between the two groups over the 24-week study period, occurring in 21% of participants in the placebo group and 19% in the probiotic group among all individuals who received study treatment (*n* = 65; see Supplementary Table S13). Gastrointestinal disorders were the most frequent adverse events occurring in 9.1% and 12.5% in the placebo and probiotic groups, respectively. One serious adverse event was recorded in the probiotic group: a participant with known recurrent idiopathic pancreatitis, with multiple episodes previous to inclusion in the study, developed pancreatitis in the study period between baseline and week 12.

## Discussion

This study is the first RCT to investigate probiotic supplementation in patients with active PsA. Over 3 months, probiotic treatment did not demonstrate superiority over placebo in achieving treatment response measured by PASDAS.

While microbiome modulation through probiotics or faecal transplantation has been associated with higher clinical remission rates in ulcerative colitis [8, 35], observational studies in other immune-mediated diseases like RA and SpA have not shown a clinical benefit to date [36]. Furthermore, the only RCT on faecal microbiometransplantation in active PsA showed less effectiveness of the intervention compared with a sham treatment regarding treatment escalation over 6 months [37].

Treatment response was lower in our study (43%) compared with the preceding pilot study (60%), possibly due to observer and reporting bias from the open-label design in the pilot, or due to the small sample size of the pilot and the resulting wider variance [12]. The placebo effect exceeded that observed in phase II/III bDMARD trials in PsA (4.1–16.1%), likely due to the inclusion of patients with MoDA rather than high disease activity (HDA) and thus a greater likelihood of reaching LDA/REM regardless of treatment [38, 39]. While including patients with HDA would have been more comprehensive, this was ruled out from the beginning, as probiotic treatment was considered most appropriate in patients with moderately active disease in which initiation or modification of immunosuppressive therapy was not immediately warranted.

PASDAS, a multidimensional composite score including joint, function and skin domains, was used in line with the preceding pilot study, on which the sample size calculation was based. At the time of the pilot, it was chosen over unidimensional scores like the DAPSA score to capture potential improvements in skin disease reported in previous probiotic studies [13, 26, 40]. However, few patients in this cohort had active skin disease at baseline, limiting the potential for detectable improvements in that domain. The components most likely to change in both treatment arms over 12 weeks were patient and physician global assessments and pain score domains, known to be influenced by non-inflammatory factors. This may further explain the high placebo response and the generally lower quantitative PASDAS values in the placebo group in the unadjusted regression analysis, which likely reflect variability in non-inflammatory components rather than a true difference in treatment effect. Adjustment for imbalanced variables at baseline (i.e. treatment, disease duration and scores for function, fatigue and depression) further reduced the group difference. In particular, pain items have shown limited structural validity for measuring inflammatory disease activity, as they are highly sensitive to other sources of pain [41, 42]. To address the susceptibility of patient-reported PASDAS domains to placebo and non-inflammatory influences, we additionally examined other disease activity measures, including SJC and CRP, as well as DAPSA and the MDA criteria. None indicated a benefit of probiotic treatment, confirming that no treatment effect was masked by the subjective PASDAS components.

Gut permeability markers were elevated in a third to half of the study participants at baseline (see Fig. 3), consistent with our pilot study, indicative of a pattern of intestinal barrier dysfunction [12]. No relevant changes on these markers were observed following probiotic administration, in contrast to mixed results reported in other RCTs [43–45]. Similarly, no relevant differences were seen in immune cell composition, or stool microbiome between probiotic and placebo after 12 weeks. Microbiome composition and diversity were not associated with treatment response. Although, bacterial dysbiosis (i.e. an alteration or disruption of the ‘normal’ microbiome as seen in healthy individuals) has been reported in patients with SpA, probiotic studies in chronic diseases associated with bacterial dysbiosis are heterogeneous and their results do not consistently demonstrate relevant microbial alteration [7, 46, 47]. In fact, in our study no relevant changes were observed in the relative abundance of *Bifidobacterium*, *Lactobacillus*, *Akkermansia* and *Ruminococcus* between baseline and week 12 neither in the placebo, nor in the probiotic group. Whether prior antibiotic treatment followed by probiotic supplementation, or a personalized probiotic formulation targeting individual microbiome dysbiosis, would induce changes in the microbiome remains unanswered. Emerging evidence suggests that responses to probiotics are highly individual: probiotic gut colonization is person-specific, governed by baseline host and microbiome features, such that identical formulations colonize some individuals but are resisted by others [48]. This has motivated interest in personalized, microbiome-guided supplementation. However, current studies have only evaluated stratifying patients by clinical phenotype such as bowel-habit subtype [49], or providing individually selected formulations without a comparator arm [50]. Whether genuinely personalized, microbiome-targeted strategies would alter clinical or microbial outcomes in general, and more specifically in PsA, remains unclear.

Limitations of this study include its single-centre design, the use of only one probiotic formulation and the relatively short duration of the blinded phase of 3 months. While the findings may not be generalizable to all probiotic supplements, the product used contained *Lactobacillus* and *Bifidobacterium* species commonly found in over-the-counter formulations [51]. This is particularly relevant given their growing use in chronic conditions despite evidence, highlighting the importance of RCTs such as this one to assess potential clinical benefits [10]. Although a longer treatment duration might have produced different results, the lack of meaningful changes from week 12 to week 24 during the open-label phase makes this unlikely. Moreover, the trial duration was consistent with that of most studies investigating probiotics in inflammatory bowel disease [8]. Due to a baseline PASDAS calculation error, five initially enrolled participants did not meet the moderate disease activity criterion and were retrospectively excluded (two in high and three in low disease activity at baseline; Fig. 1). Although this error was identified after unblinding, the exclusion was based on an objective, prespecified eligibility criterion (baseline PASDAS) that did not influence treatment allocation. Post-randomization exclusions based on such objective criteria have been considered acceptable, as wrongly included patients can introduce random error by responding differently to treatment compared with the population under investigation [49]. Including all five participants regardless of their baseline PASDAS would have yielded a treatment response of 60% (18/30) in the placebo vs 45% (14/31) in the probiotic group, consistent with the primary analysis [52]. Furthermore, the exclusion of the five participants did not compromise the validity of our results, as the recruitment exceeded the required sample size, providing a power buffer.

Some numbers in this article differ from those in our earlier ACR 2023 conference abstract. These differences are mainly attributable to the resulting change in the analysed population. Both abstract and final article show no benefit of the probiotic over placebo [53].

The unexpectedly high placebo response (65% vs the 20% assumed) means we cannot exclude a type II error for small treatment effects. However, none of the point estimates for the primary outcome or quantitative PASDAS indicated a trend towards probiotic benefit, and this was preserved across all sensitivity analyses, with the upper confidence bound for the primary outcome (OR 0.4, 95% CI: 0.13–1.18) making a relevant probiotic effect unlikely.

Studies show that the gut microbiome is shaped less by host genetics than by factors related to diet, drugs and anthropometric measurements [54]. A Mediterranean diet and exercise have been associated with lower disease activity in PsA [55], and EULAR recommendations emphasize the importance of a healthy, balanced diet and physical activity in patients with rheumatic diseases in regard to disease activity. In our study, most dietary factors, BMI and alcohol consumption were broadly comparable between the groups, with modest differences in fish and sweet-dessert intake. However, we did not specifically assess physical activity, which could have provided additional information [56].

Given the randomized design, allocation concealment, blinding of patients and investigators, and lack of identifiable attrition bias, we consider the overall risk of bias to be low. Furthermore, sensitivity analyses including multivariable regression analyses to adjust for potential confounders, multiple imputation for remaining missing data, a per-protocol analysis and an analysis taking into account treatment adherence, by analysing only participants in the intervention arm with probiotic bacterial strain-specific DNA in the stool, showed no differences in outcomes, supporting the robustness of our findings. Finally, although no relevant safety signals were observed, the study was neither large nor long enough to assess rarer adverse events.

In conclusion, this RCT in PsA with moderately active disease did not show a clinical benefit of the tested multispecies probiotic treatment compared with placebo regarding treatment response after 3 months of treatment under the conditions studied.

## Supplementary Material

keag547_Supplementary_Data

## Data Availability

The 16S rRNA gene amplicon sequencing data have been deposited at the Sequence Read Archive under the BioProject accession PRJNA1374100. Deidentified individual participant data underlying the results reported in this article will be made available upon reasonable request to the corresponding authors.
