# Supplementary material for: Efficacy and safety of probiotic supplementation in PsA—a randomized controlled trial
Source: Rheumatology (Oxford). 2026 Oct 5;65(10):keag547. doi: 10.1093/rheumatology/keag547 (PMC13656753; doi:10.1093/rheumatology/keag547)
Supplement: Supplementary file 1 [file keag547_supplementary_data.docx]

# Supplementary material

## Supplementary Data S1:

**Composition of the probiotic and placebo formulation**

*Probiotic:*

Corn starch, maltodextrin, inulin, potassium chloride, plant protein (rice), bacterial strains, magnesium sulfate, fructooligosaccharides (FOS), enzymes (amylases), manganese sulfate*

*9 human derived bacterial strains with at least 7.5 billion CFU (colony-forming units) per 1 portion (= 3 g) and 15 billion CFU per 2 portions (= 6 g):

- *Lactobacillus casei*
- *Lactobacillus acidophilus*
- *Lactobacillus paracasei*
- *Bifidobacterium lactis*
- *Lactobacillus salivarius*
- *Lactococcus lactis*
- *Bifidobacterium lactis*
- *Lactobacillus plantarum*
- *Bifidobacterium bifidum*

The specific strain designations (e.g., strain numbers) cannot be disclosed due to contractual agreements.

*Placebo:*

Corn starch, maltodextrin, potassium chloride, magnesium sulfate, manganese sulfate

## Supplementary Data S2:

**Details on methods gut permeability markers, phagocytosis tests and flow cytometry**

*Gut permeability markers*

Stool samples were collected from participants using sterile collection containers. Participants were instructed to collect a spontaneous bowel movement, avoiding contamination with urine or water, and to refrain from using laxatives, enemas, or suppositories for at least 48 hours before sampling. Samples were stored frozen at −80 °C to wait for completion of recruitment. Specimens were shipped in insulated transport kits with frozen gel packs to maintain the cold chain during transit.

Quantitative determination of stool zonulin, alpha-1 antitrypsin, and calprotectin was performed by Biovis’ Diagnostik according to their validated standard operating procedures. In brief, all three biomarkers were measured using commercially available enzyme-linked immunosorbent assay (ELISA) kits (Immundiagnostik AG, Bensheim, Germany). Stool samples were homogenized in extraction buffer, centrifuged to remove particulate matter, and the resulting supernatants were assayed in duplicate. Optical density was recorded at the appropriate wavelength using a microplate reader, and analyte concentrations were calculated from standard curves generated from known calibrators. Laboratory staff were blinded to participant group allocation during the analyses and the samples were shipped using randomized nomenclature.

*Phagocytosis tests*

Oxidative burst and phagocytosis were examined using commercially available test kits (Phagoburst® and Phagotest®, BD Biosciences). Assays were performed according to the manufacturer’s protocol. In brief, heparinized whole blood was incubated with opsonized FITClabelled E.coli suspension. Phagocytosis is indicated by FITC-positivity of granulocytes and monocytes measured flow cytometry. Formation of reactive oxidants in granulocytes and monocytes during the oxidative burst weremonitored by the addition and oxidation of DHR 123 in flow cytometry.

*Flow cytometry*

Peripheral blood mononuclear cells (PBMCs) were isolated by Histopaque density gradient centrifugation. For flow cytometry, approximately 1 × 10^6, PBMCs were stained with a master mix of fluorochrome-conjugated antibodies for 30 min at 4°C in the dark, washed twice with staining buffer (BD biosciences), and acquired on a BD Lyric (BD Biosciences). Compensation was performed using single-stain compensation beads and data analyzed in FlowJo v10 (BD). Antibody markers and are provided below.

| **T-cell panel** |  |  |
| --- | --- | --- |
| **Population identified** | **Cell Tupe** | **Marker** |
| General back bone | T-cell | CD3+ |
|  | CD4 T-cells | CD4+ CD8- |
|  | CD8 T-cells | CD4- CD8+ |
| T-helper subsets | Th1 | CXCR3+ CCR4- CCR6- |
|  | Th2 | CXCR3- CCR4+ CCR6- |
|  | Th17 | CXCR3- CCR4- CCR6+ |
|  | Th1/17 | CXCR3+ CCR4- CCR6+ |
|  | T-reg | CD25 + CD127 - |
| Effector/memory populations | Effector memory | CCR7-CD45RA- |
|  | Central memory | CCR7+CD45RA- |
|  | Naïve | CCR7+CD45RA+ |
|  | Effector | CCR7-CD45RA+ |

## Supplementary Data S3:

**Details on methods on microbiome analysis and probiotic strain detection**

*Microbiome analysis*

DNA was extracted from 56 stool samples representative of probiotics and placebo experimental groups at baseline and after 12 weeks of intervention with the QIAamp Fast DNA Stool Mini Kit (Qiagen, Germany) following the manufacturer's instructions. The V4 hypervariable region of the bacterial and archaeal 16S rRNA gene was amplified using modified primers 515F/806R.(1, 2) DNA extraction, sequencing, and raw data processing was performed at the Joint Microbiome Facility of the Medical University of Vienna and the University of Vienna (project ID JMF-2212-15) as described previously,(3) and sequenced on a Illumina MiSeq (2 x 300bp). Amplicon pools were extracted from the raw sequencing data using the FASTQ workflow in BaseSpace (Illumina) with default parameters. Raw data processing was performed as described previously.(3) Demultiplexing was performed with the python package demultiplex (Laros JFJ, github.com/jfjlaros/demultiplex) allowing one mismatch for barcodes and two mismatches for linkers and primers. Amplicon sequence variants (ASVs) were inferred using the DADA2 R package v1.42 applying the recommended workflow.(4, 5) FASTQ reads 1 and 2 were trimmed at 220 nt and 150 nt with allowed expected errors of 2. ASV sequences were subsequently classified using DADA2 and the SILVA database SSU Ref NR 99 release 138.1 using a confidence threshold of 0.5. ASVs without classification or classified as eukaryotes, mitochondria, or chloroplasts, as well as well-known buffer contaminations were removed.(6, 7) No further contaminants were detected, the DNA extraction controls (n=5) contained between 2-19 sequence reads, affiliated to known buffer contaminants. After filtering, only samples with at least 1000 read pairs were kept for further analyses, resulting in the exclusion of 3 samples. Sequencing depth of the remaining dataset ranged from 1051 to 41 294 reads per sample. Downstream analyses were performed using R v4.3.2 and Bioconductor v3.16 packages SummarizedExperiment v1.32, SingleCellExperiment v1.24, TreeSummarizedExperiment v2.8,(8) mia v1.8,(9) vegan v2.6-4,(10) phyloseq v1.44,(11) microbiome v1.22 and microViz v0.10.8.(12, 13) Alpha diversity (i.e. richness and diversity indexes) was calculated on rarified data (1051 read pairs) using R packages vegan and mia. Beta diversity was calculated by performing a PCoA using the Aitchison distance, with R package microViz. The difference in per-group centroids was tested with a PERMANOVA on Aitchison distances using R packages vegan and microViz.

*qRT-PCR analysis for probiotic strain detection in stool samples*

The 56 stool samples were furthermore tested for the presence of any of probiotic strains, that were applied with the product, by quantitative real time PCR (qRT-PCR) to confirm adherence of probands to the study protocol. The primer/probe sets were specific enough to detect strains in samples from the probiotic group. Quantify the DNA concentration was performed by using DeNovix spectrophotometer (Wilmington, DE 19810 USA). Primers and probes used were designed and validated to every single strain of the probiotic formulation and they do not form any cross-reactions. Several DNA samples from the placebo group were used to determine non-specific binding of the primer/probe sets. A cycle threshold (CT) of 35 was set as the cutoff for positive detection of at least one of the probiotic strains. The exact primer and probe sequences cannot be disclosed due to contractual obligations.

QuantStudio5 PCR machine and consumables of Thermo Scientific (63303 Dreieich Deutschland) were used for the PCR reaction in triplicates. All samples were tested for the presence of bacterial DNA using universal 16S region primer sets in DNA material of placebo and probiotic samples using a standard PCR protocol. The strain specific primer/probe sets were used to detect the applied probiotic strains in the samples from the probiotic group using a 2-step PCR protocol with an annealing temperature of 60°C.

## Supplementary figure S1:

**Flowchart according to per protocol analysis**

HDA, high disease activity; LDA, low disease activity

## Supplementary figure S2:

Chao and Shannon index depicting alpha diversity. Differences between groups were assessed by performing a non-parametric Wilcox-test. No significant differences were found.

## Supplementary figure S3:

Microbial community structure between groups, as assesssed by a PcoA using Aitchinson distance. Statistical differences between groups were assessed by a PERMANOVA (p>0.05).

## Supplementary figure S4:

Relative abundance of selected genera at baseline and week 12 between the probiotic and placebo group.

## Supplementary table S1:

**Baseline characteristics according to per protocol analysis**

| **Characteristic** | **Probiotic** N = 28 | **Placebo** N = 25 | **SMD** |
| --- | --- | --- | --- |
| Age, years | 52 (11) | 48 (14) | 0.36 |
| Female | 18 (64%) | 15 (60%) | 0.09 |
| BMI, kg/m² | 28.2 (5.9) | 27.6 (4.5) | 0.11 |
| Current smoker | 7 (25%) | 1 (4.2%) | 0.62 |
| Consumes alcohol regularly | 18 (64%) | 17 (71%) | -0.14 |
| Vegetarian | 0 (0%) | 2 (8.0%) | -0.42 |
| Disease duration, months | 91 (0 to 384) | 23 (0 to 421) | 0.14 |
| PASDAS | 3.9 (3.2 to 5.0) | 3.8 (3.3 to 4.9) | 0.08 |
| DAPSA | 12.8 (8.1 to 25.5) | 11.5 (8.3 to 22.0) | 0.47 |
| MDA | 3 (14%) | 4 (20%) | -0.17 |
| Tender joint count | 3.0 (1.0 to 14.0) | 2.0 (0.0 to 11.0) | 0.45 |
| Swollen joint count | 1.0 (0.0 to 5.0) | 1.0 (0.0 to 5.0) | -0.13 |
| Pain visual analog scale | 35 (10 to 80) | 40 (30 to 50) | 0.17 |
| Patient global assessment | 30 (10 to 80) | 40 (30 to 60) | 0.18 |
| Evaluator global assessment | 20 (0 to 40) | 25 (20 to 40) | -0.37 |
| Leeds Enthesitis Index | 0 (0 to 2) | 0 (0 to 4) | -0.09 |
| Dactylitis count | 0 (0 to 0) | 0 (0 to 1) | -0.29 |
| C-reactive protein, mg/L | 2 (1 to 48) | 2 (0 to 10) | 0.50 |
| Erythrocyte sedimentation rate, mm/h | 11 (2 to 27) | 6 (1 to 31) | 0.37 |
| SF-36 PCS Score | 39 (20 to 47) | 39 (21 to 55) | -0.39 |
| PSAID-12 Score | 2.9 (0.6 to 7.4) | 2.6 (0.3 to 4.8) | 0.44 |
| HADS-D Depression Score | 7.0 (0.0 to 20.0) | 4.0 (0.0 to 14.0) | 0.61 |
| HADS-D Anxiety Score | 7.5 (0.0 to 20.0) | 7.0 (0.0 to 13.0) | 0.53 |
| PASI Score | 0.0 (0.0 to 6.5) | 0.0 (0.0 to 6.0) | 0.15 |
| FACIT Fatigue Score | 30 (8 to 51) | 38 (13 to 52) | -0.57 |
| HAQ Score | 1.00 (0.00 to 1.88) | 0.25 (0.00 to 1.25) | 1.2 |
| Biologic DMARD |  |  | 0.55 |
| None | 12 (43%) | 14 (56%) |  |
| TNFi | 9 (32%) | 6 (24%) |  |
| IL17i | 5 (18%) | 4 (16%) |  |
| IL23i | 2 (7.1%) | 0 (0%) |  |
| IL12+23i | 0 (0%) | 1 (4.0%) |  |
| Conventional DMARD |  |  | 0.62 |
| None | 20 (71%) | 18 (72%) |  |
| MTX | 7 (25%) | 4 (16%) |  |
| LFN | 0 (0%) | 3 (12%) |  |

| Targeted synthetic DMARD |  |  | 0.14 |
| --- | --- | --- | --- |
| None | 23 (82%) | 21 (84%) |  |
| JAKi | 2 (7.1%) | 1 (4.0%) |  |
| Apremilast | 3 (11%) | 3 (12%) |  |
| Current NSAID intake | 15 (60%) | 7 (37%) | 0.48 |
| BMI: Body Mass Index; CRP: C-reactive protein; DAPSA: Disease Activity in Psoriatic Arthritis; DMARD: Disease-Modifying Antirheumatic Drug; FACIT: Functional Assessment of Chronic Illness Therapy; HAQ: Health Assessment Questionnaire; HADS-D: Hospital Anxiety and Depression Scale - Depression; IL12+23i: Interleukin 12 and 23 inhibitor; IL17i: Interleukin 17 inhibitor; IL23i: Interleukin 23 inhibitor; JAKi: Janus Kinase inhibitor; LFN: Leflunomide; MDA: Minimal Disease Activity; MTX: Methotrexate; NSAID: Non-Steroidal Anti-Inflammatory Drug; PASI: Psoriasis Area and Severity Index; PASDAS: Psoriatic Arthritis Disease Activity Score; PSAID-12: Psoriatic Arthritis Impact of Disease - 12 items; SF-36 PCS: Short Form 36 Physical Component Summary; SMD: Standardized Mean Difference; TNFi: Tumor Necrosis Factor inhibitor.  Mean (SD), Median (Min to Max) or n (%)  Missing values: BMI was available for 48 (91%) of patients; Erythrocyte sedimentation rate for 52 (98%) of patients; Current smoker status for 52 (98%) of patients; Regular alcohol consumption for 52 (98%) of patients; Vegetarian status for 52 (98%) of patients; PSAID-12 Score for 52 (98%) of patients; PASI Score for 45 (85%) of patients; HAQ Score for 50 (94%) of patients; MDA for 42 (79%) of patients and Current NSAID intake 44 (83%) of patients.  This includes 53 patients according to the per protocol complete case analysis. | | | |

Supplementary Table 2:

**Dietary intake at baseline**

|  | **Baseline** | | | **Week 12** | | |
| --- | --- | --- | --- | --- | --- | --- |
| **Characteristic** | **Probiotic** N = 28 | **Placebo** N = 26 | **SMD** | **Probiotic** N = 29 | **Placebo** N = 22 | **SMD** |
| Diet type |  |  | 0.41 |  |  | 0.31 |
| Omnivore | 28 (100%) | 24 (92%) |  | 29 (100%) | 21 (95%) |  |
| Vegetarian/Vegan | 0 (0%) | 2 (7.7%) |  | 0 (0%) | 1 (4.5%) |  |
| Sugary drinks (glasses/day) | 1 (0 to 5) | 1 (0 to 5) | 0.16 | 1 (0 to 6) | 0 (0 to 6) | 0.24 |
| Wholegrain products (%) | 35 (3 to 100) | 50 (2 to 80) | -0.09 | 50 (0 to 100) | 50 (20 to 95) | -0.01 |
| Fruit (portions/day) | 1 (0 to 3) | 1 (0 to 2) | 0.22 | 1 (0 to 3) | 1 (0 to 3) | -0.01 |
| Vegetables (portions/day) | 1 (0 to 3) | 1 (0 to 3) | 0.12 | 1 (0 to 3) | 1 (0 to 3) | 0.07 |
| Red meat (per week) | 1 (0 to 2) | 1 (0 to 2) | 0.26 | 1 (0 to 2) | 1 (0 to 2) | 0.23 |
| Processed meat (per week) | 1 (0 to 2) | 1 (0 to 2) | 0.06 | 1 (0 to 2) | 1 (0 to 2) | 0.22 |
| Fish (per week) | 2 (0 to 3) | 1 (0 to 2) | 0.51 | 1 (0 to 3) | 1 (0 to 2) | 0.13 |
| Sweets/desserts (frequency) | 1 (0 to 3) | 2 (0 to 4) | -0.39 | 1 (0 to 3) | 2 (0 to 3) | -0.31 |
| Diet type: usual dietary pattern, dichotomised as omnivore vs. vegetarian/vegan (original 5-level scale: omnivore / ovo- / lacto- / ovo-lacto-vegetarian / vegan). Sugary drinks: sweetened beverages per day in glasses (~200 ml each). Wholegrain products: self-reported share of wholegrain vs. white-flour products (%). Fruit and Vegetables: portions per day (0 = <1; 1 = 1; 2 = 2; 3 = >2; 1 portion ≈ 1 handful). Red meat: pork/beef/veal/lamb excluding processed meat, per week (0 = none; 1 = 1–2; 2 = >2). Processed meat: per week (0 = none; 1 = 1–2; 2 = ≥3). Fish: per week (0 = none; 1 = <1; 2 = 1–2; 3 = >2). Sweets/desserts: frequency (0 = never/rarely; 1 = 1–3/week; 2 = 4–6/week; 3 = 1/day; 4 = several/day). SMD: standardised mean difference (between probiotic and placebo). | | | | | | |

## Supplementary table S3:

**Sensitivity analyses of chi square statistics for primary outcome at week 12**

| **Analysis** | **Probiotic (n/N)** | **Placebo (n/N)** | **χ²** | **df** | **p-value** |
| --- | --- | --- | --- | --- | --- |
| PP | 12/28 | 16/25 | 1.60 | 1 | 0.206 |
| ITT MI | 31 | 29 | 1.28 | 1 | 0.258 |
| Carrier | 9/19 | 17/26 | 0.82 | 1 | 0.367 |
| ITT MI, Intention-to-treat analysis using multiple imputation; n/N, Number of participants reaching the treatment target (n) out of all analyzed (N); PP, Per-protocol analysis. | | | | | |
| Carrier includes a subset of the ITT complete case population in which probiotic strains were detected in the probiotic group using qPCR. | | | | | |
| For ITT MI, only total group sizes are shown as the multiple imputation model pools estimates and variances from several imputed datasets with different numbers of treatment responses and then derives the test statistic. | | | | | |

## Supplementary table S4:

**Sensitivity analyses of treatment response and PASDAS at Week 12**

| **Outcome** | **Population** | **N Probiotic** | **n (%) Probiotic$** | **N Placebo** | **n (%) Placebo$** | **OR (95% CI)** | **Effect size (95% CI)** |
| --- | --- | --- | --- | --- | --- | --- | --- |
| Treatment response* | ITT MI | 31 | NA | 29 | NA | 0.45 (0.14 to 1.41) | NA |
| Treatment response** | ITT MI | 31 | NA | 29 | NA | 0.89 (0.18 to 4.42) | NA |
| Treatment response* | PP | 28 | 12 (42.9%) | 25 | 16 (64%) | 0.42 (0.14 to 1.26) | NA |
| Treatment response* | Carrier | 19 | 9 (47.4%) | 26 | 17 (65.4%) | 0.48 (0.14 to 1.58) | NA |
| PASDAS* | ITT MI | 31 | NA | 29 | NA | NA | 0.7 (0.03 to 1.37) |
| PASDAS** | ITT MI | 31 | NA | 29 | NA | NA | 0.12 (-0.78 to 1.02) |
| PASDAS* | PP | 28 | NA | 25 | NA | NA | 0.7 (0.05 to 1.35) |
| PASDAS* | Carrier | 19 | NA | 26 | NA | NA | 0.44 (-0.27 to 1.15) |
| MDA* | ITT CC | 26 | 6 (23.1%) | 21 | 14 (66.7%) | 0.15 (0.04 to 0.52) | NA |
| MDA** | ITT CC | 22 | 6 (27.3%) | 15 | 9 (60%) | 0.5 (0.05 to 4.2) | NA |
| DAPSA* | ITT CC | 30 | NA | 26 | NA | NA | 3.02 (-0.56 to 6.59) |
| DAPSA** | ITT CC | 25 | NA | 19 | NA | NA | -0.61 (-5.54 to 4.32) |
| Swollen joint count* | ITT CC | 30 | NA | 26 | NA | NA | -0.35 (-1.09 to 0.39) |
| Swollen joint count** | ITT CC | 25 | NA | 19 | NA | NA | -1 (-1.99 to 0) |
| C-reactive protein* | ITT CC | 30 | NA | 26 | NA | NA | 1.05 (-1.4 to 3.5) |
| C-reactive protein** | ITT CC | 25 | NA | 19 | NA | NA | 1.39 (-2.3 to 5.08) |

In the probiotic group, DNA for qPCR was unavailable for 7 of 30 participants, and bacterial strains were undetectable in 4. In the placebo group, probiotic bacterial strains were not detected in any participant.

Effect sizes and odds ratios refer to the difference between the probiotic and placebo groups.

Treatment response: OR < 1 indicates lower odds of response in the probiotic group compared to placebo. PASDAS: Positive effect sizes indicate higher scores in the probiotic group compared to placebo.

*univariable model for group assignment (probiotic or placebo)

**multivariable model for group assignment adjusting for DMARD treatment, NSAID treatment, disease duration, Health Assessment Questionnaire (HAQ) score, Functional Assessment of Chronic Illness Therapy (FACIT) fatigue score, and Hospital Anxiety and Depression Scale - Depression (HADS-D) score.

$number and % of participants achieving treatment response. This is not given for the multiple imputation model as the model pools odds ratios derived from several imputed datasets with different numbers of treatment responses.

ITT CC, modified intention to treat complete case analysis; ITT MI, intention to treat multiple imputations; OR, odds ratio; PASDAS, Psoriatic Arthritis Disease Acitivity Score; PP, per protocol Carrier, carrier of probiotic strand assessed by quantitative polymerase chain reaction (qPCR):

## Supplementary table S5:

**Clinical Outcomes at week 12 according to the per protocol analysis**

|  | **Probiotic** | | **Placebo** | |
| --- | --- | --- | --- | --- |
| **Outcome** | **N** | **Median (Min to Max)** | **N** | **Median (Min to Max)** |
| PASDAS | 28 | 3.2 (0.5 to 5.7) | 25 | 2.8 (0.3 to 4.9) |
| Tender joint count | 28 | 2 (0 to 13) | 25 | 1 (0 to 11) |
| Swollen joint count | 28 | 0 (0 to 7) | 25 | 0 (0 to 4) |
| Pain visual analog scale | 28 | 30 (10 to 90) | 25 | 20 (0 to 60) |
| Patient global assessment | 28 | 29.5 (0 to 90) | 25 | 20 (0 to 50) |
| Evaluator global assessment | 28 | 19 (0 to 80) | 25 | 10 (0 to 50) |
| Leeds Enthesitis Index | 28 | 0 (0 to 3) | 25 | 0 (0 to 0) |
| Dactylitis count | 28 | 0 (0 to 0) | 25 | 0 (0 to 0) |
| C-reactive protein | 28 | 1.8 (0 to 16.7) | 25 | 1.9 (0.4 to 14.9) |
| Erythrocyte sedimentation rate | 27 | 7 (2 to 19) | 24 | 6.5 (2 to 23) |
| SF-36 PCS Score | 28 | 37.7 (22 to 53.1) | 25 | 45.6 (24.8 to 57.6) |
| PSAID-12 Score | 25 | 2.8 (0.5 to 7.9) | 25 | 1.3 (0 to 4.5) |
| HADS-D Depression Score | 28 | 4.5 (1 to 19) | 25 | 3 (0 to 11) |
| HADS-D Anxiety Score | 28 | 7 (0 to 18) | 25 | 5 (0 to 13) |
| PASI Score | 24 | 0 (0 to 3.8) | 21 | 0 (0 to 2.7) |
| FACIT Fatigue Score | 28 | 36 (4 to 50) | 25 | 43 (17 to 52) |
| HAQ Score | 28 | 0.8 (0 to 2.8) | 24 | 0.4 (0 to 1.5) |
| N represents the number of patients with available data per group for each outcome, according to the per protocol analysis. | | | | |
| FACIT, Functional Assessment of Chronic Illness Therapy; HAQ, Health Assessment Questionnaire; HADS-D, Hospital Anxiety and Depression Scale; PASI, Psoriasis Area and Severity Index; PASDAS, Psoriatic Arthritis Disease Activity Score; PSAID-12, Psoriatic Arthritis Impact of Disease - 12 items; SF-36 PCS, Short Form 36 Physical Component Summary. | | | | |

## Supplementary table S6:

**Clinical outcomes at week 12 and week 24 in the Placebo Group**

| **Outcome (Timepoint)** | **N** | **Median (Min to Max)** |
| --- | --- | --- |
| PASDAS (W12) | 23 | 2.7 (0.3 to 4.6) |
| PASDAS (W24) | 23 | 2.8 (0.5 to 4.6) |
| Tender joint count (W12) | 23 | 1 (0 to 11) |
| Tender joint count (W24) | 23 | 0 (0 to 5) |
| Swollen joint count (W12) | 23 | 0 (0 to 4) |
| Swollen joint count (W24) | 23 | 0 (0 to 3) |
| Pain visual analog scale (W12) | 23 | 20 (0 to 60) |
| Pain visual analog scale (W24) | 23 | 20 (0 to 70) |
| Patient global assessment (W12) | 23 | 20 (0 to 50) |
| Patient global assessment (W24) | 23 | 20 (0 to 70) |
| Evaluator global assessment (W12) | 23 | 10 (0 to 50) |
| Evaluator global assessment (W24) | 23 | 10 (0 to 30) |
| Leeds Enthesitis Index (W12) | 23 | 0 (0 to 0) |
| Leeds Enthesitis Index (W24) | 23 | 0 (0 to 1) |
| Dactylitis count (W12) | 23 | 0 (0 to 0) |
| Dactylitis count (W24) | 23 | 0 (0 to 0) |
| C-reactive protein (W12) | 23 | 1.9 (0.4 to 14.9) |
| C-reactive protein (W24) | 23 | 1.5 (0.1 to 11.9) |
| Erythrocyte sedimentation rate (W12) | 22 | 6 (2 to 16) |
| Erythrocyte sedimentation rate (W24) | 23 | 6 (2 to 64) |
| SF-36 PCS Score (W12) | 23 | 45.7 (24.8 to 57.6) |
| SF-36 PCS Score (W24) | 23 | 44.4 (20.8 to 54.8) |
| PSAID-12 Score (W12) | 23 | 1.3 (0 to 4.5) |
| PSAID-12 Score (W24) | 23 | 1.4 (0.2 to 7.6) |
| HADS-D Depression Score (W12) | 23 | 3 (0 to 11) |
| HADS-D Depression Score (W24) | 23 | 2 (0 to 12) |
| HADS-D Anxiety Score (W12) | 23 | 5 (0 to 13) |
| HADS-D Anxiety Score (W24) | 23 | 3 (0 to 13) |
| PASI Score (W12) | 20 | 0 (0 to 2.7) |
| PASI Score (W24) | 16 | 0 (0 to 2) |
| FACIT Fatigue Score (W12) | 23 | 43.3 (17 to 52) |
| FACIT Fatigue Score (W24) | 23 | 45 (16 to 52) |
| HAQ Score (W12) | 23 | 0.4 (0 to 1.5) |
| HAQ Score (W24) | 22 | 0.4 (0 to 1.8) |
| N represents the number of patients with available data per outcome at each time point. This includes 23 placebo-randomized patients who received probiotic from Week 12–24 (open-label phase), per modified intention to treat complete case analysis. | | |
| FACIT, Functional Assessment of Chronic Illness Therapy; HAQ, Health Assessment Questionnaire; HADS-D, Hospital Anxiety and Depression Scale; PASI, Psoriasis Area and Severity Index; PASDAS, Psoriatic Arthritis Disease Activity Score; PSAID-12, Psoriatic Arthritis Impact of Disease – 12 items; SF-36 PCS, Short Form 36 Physical Component Summary, W12, week 12; W24, week 24. | | |

## Supplementary table S7:

**Gut permeability markers at baseline and Week 12**

|  | **Probiotic** | | **Placebo** | |
| --- | --- | --- | --- | --- |
| **Outcome** | **N** | **Median (Min to Max)** | **N** | **Median (Min to Max)** |
| Zonulin (BSL) | 28 | 57 (24 to 117) | 25 | 57 (12 to 104) |
| Zonulin (W12) | 29 | 51 (23 to 126) | 25 | 47 (12 to 114) |
| Calprotectin (BSL) | 30 | 31 (18 to 390) | 24 | 32 (18 to 232) |
| Calprotectin (W12) | 29 | 32 (18 to 301) | 25 | 32 (18 to 196) |
| Alpha-1 Antitrypsin (BSL) | 30 | 26 (2 to 77) | 24 | 25 (2 to 138) |
| Alpha-1 Antitrypsin (W12) | 30 | 30 (2 to 81) | 25 | 28 (2 to 162) |
| Samples were measured in duplicates by ELISA at Baseline and Week 12. Only values with ≤20% deviation between duplicates were retained. | | | | |
| N represents the number of patients with available data according to the modified intention to treat complete case analysis. | | | | |
| Normal reference ranges: Zonulin: <55 ng/ml; Calprotectin: <50 mg/l; Alpha-1 Antitrypsin: <27.5 mg/dl. | | | | |
| BSL, baseline; W12, week 12. | | | | |

## Supplementary table S8:

**Gut permeability at Week 12 and Week 24**

| **Outcome** | **N** | **Median (Min to Max)** |
| --- | --- | --- |
| Zonulin (W12) | 22 | 50 (12 to 114) |
| Zonulin (W24) | 20 | 49 (14 to 229) |
| Calprotectin (W12) | 22 | 34 (18 to 196) |
| Calprotectin (W24) | 22 | 32 (18 to 203) |
| Alpha-1 Antitrypsin (W12) | 22 | 30 (2 to 162) |
| Alpha-1 Antitrypsin (W24) | 21 | 24 (3 to 88) |
| Samples were measured in duplicates by ELISA at Week 12 and Week 24. Only values with ≤20% deviation between duplicates were retained. | | |
| N represents the number of patients with available data. Includes 23 placebo-randomized patients who received probiotic from Week 12–24, per ITT complete case analysis. | | |
| Normal reference ranges: Zonulin: <55 ng/ml; Calprotectin: <50 mg/l; Alpha-1 Antitrypsin: <27.5 mg/dl. | | |
| BSL, baseline; W12, week 12. | | |

## Supplementary table S9

**Absolute Immune Cell Counts at Baseline and Week 12**

|  | **Probiotic** | | **Placebo** | |
| --- | --- | --- | --- | --- |
|  | **N** | **Median (Min to Max)** | **N** | **Median (Min to Max)** |
| CD4 (BSL) | 29 | 911.00 (468.00 to 1693.00) | 26 | 763.00 (382.00 to 1464.00) |
| CD4 (W12) | 30 | 945.50 (343.00 to 1489.00) | 26 | 854.00 (367.00 to 1540.00) |
| Th1 (BSL) | 27 | 297.47 (31.36 to 783.86) | 24 | 236.18 (116.53 to 606.48) |
| Th1 (W12) | 25 | 307.29 (78.20 to 648.66) | 23 | 248.72 (110.83 to 639.39) |
| Th1-17 (BSL) | 27 | 3.34 (0.56 to 31.97) | 24 | 3.09 (0.36 to 29.68) |
| Th1-17 (W12) | 27 | 2.78 (1.16 to 14.67) | 24 | 2.83 (0.13 to 9.29) |
| Th2 (BSL) | 26 | 27.50 (7.99 to 94.74) | 23 | 29.13 (8.33 to 111.94) |
| Th2 (W12) | 27 | 30.77 (10.84 to 101.58) | 25 | 26.81 (2.25 to 138.75) |
| Th17 (BSL) | 27 | 21.69 (4.37 to 96.86) | 24 | 19.60 (2.35 to 82.05) |
| Th17 (W12) | 27 | 20.13 (4.87 to 72.25) | 25 | 21.78 (2.39 to 83.62) |
| Treg (BSL) | 27 | 52.23 (24.28 to 140.86) | 26 | 49.72 (18.88 to 120.09) |
| Treg (W12) | 27 | 52.02 (23.42 to 151.51) | 26 | 53.56 (13.60 to 166.37) |
| CD4 CM (BSL) | 27 | 294.83 (121.68 to 768.62) | 26 | 235.51 (103.14 to 479.61) |
| CD4 CM (W12) | 27 | 311.64 (119.71 to 564.60) | 26 | 238.80 (70.83 to 508.16) |
| CD4 Eff (BSL) | 27 | 16.97 (4.74 to 101.53) | 26 | 15.16 (4.27 to 133.00) |
| CD4 Eff (W12) | 27 | 16.39 (5.82 to 90.26) | 26 | 14.06 (4.96 to 192.05) |
| CD4 EM (BSL) | 27 | 218.90 (89.39 to 553.61) | 26 | 186.05 (92.45 to 476.62) |
| CD4 EM (W12) | 27 | 247.15 (118.18 to 551.99) | 26 | 182.71 (94.46 to 499.00) |
| CD4 Naive (BSL) | 28 | 297.01 (0.00 to 931.17) | 26 | 312.04 (117.73 to 917.93) |
| CD4 Naive (W12) | 27 | 302.35 (82.92 to 879.77) | 26 | 326.94 (106.38 to 877.80) |
| CD8 (BSL) | 29 | 329.00 (159.00 to 955.00) | 26 | 350.50 (82.00 to 767.00) |
| CD8 (W12) | 30 | 450.00 (104.00 to 906.00) | 26 | 401.50 (125.00 to 709.00) |
| CD8 CM (BSL) | 27 | 19.45 (3.24 to 101.95) | 26 | 18.29 (3.73 to 130.20) |
| CD8 CM (W12) | 27 | 25.53 (2.26 to 102.83) | 26 | 25.17 (3.34 to 155.36) |
| CD8 Eff (BSL) | 27 | 99.67 (7.57 to 429.75) | 26 | 73.10 (4.97 to 401.29) |
| CD8 Eff (W12) | 27 | 112.63 (6.81 to 453.47) | 26 | 72.93 (8.69 to 316.24) |
| CD8 EM (BSL) | 27 | 138.60 (37.06 to 461.38) | 26 | 130.94 (2.31 to 255.68) |
| CD8 EM (W12) | 27 | 160.70 (53.38 to 427.20) | 26 | 117.86 (4.88 to 289.91) |
| CD8 Naive (BSL) | 27 | 66.67 (19.71 to 259.92) | 26 | 96.98 (22.53 to 421.85) |
| CD8 Naive (W12) | 27 | 76.16 (16.42 to 247.78) | 26 | 68.36 (27.09 to 349.75) |
| Absolute immune cell counts (cells/μL) measured by flow cytometry. | | | | |
| N indicates the number of patients with available data at each timepoint according to the modified intention-to-treat complete case analysis. | | | | |
| CM, Central memory; Eff, Effector; Treg, Regulatory T cells. | | | | |

## Supplementary table S10

**Absolute Immune Cell Counts at Week 12 and the Difference to Week 24**

|  | **N** | **Median (Min to Max)** |
| --- | --- | --- |
| CD4 (W12) | 23 | 902.00 (450.00 to 1540.00) |
| CD4 (W24) | 22 | 871.50 (483.00 to 1426.00) |
| Th1 (W12) | 20 | 250.25 (131.70 to 639.39) |
| Th1 (W24) | 19 | 252.22 (105.67 to 692.31) |
| Th2 (W12) | 22 | 25.32 (2.25 to 138.75) |
| Th2 (W24) | 21 | 28.16 (1.42 to 151.36) |
| Th17 (W12) | 22 | 22.36 (4.49 to 83.62) |
| Th17 (W24) | 21 | 26.96 (3.46 to 53.39) |
| Treg (W12) | 23 | 52.50 (13.60 to 103.29) |
| Treg (W24) | 21 | 44.42 (18.23 to 113.45) |
| CD4 CM (W12) | 23 | 254.87 (91.12 to 508.16) |
| CD4 CM (W24) | 21 | 247.06 (110.26 to 748.65) |
| CD4 Eff (W12) | 23 | 12.15 (4.96 to 124.52) |
| CD4 Eff (W24) | 21 | 15.21 (2.15 to 102.79) |
| CD4 EM (W12) | 23 | 184.96 (94.46 to 499.00) |
| CD4 EM (W24) | 21 | 204.83 (74.62 to 546.16) |
| CD4 Naive (W12) | 23 | 333.74 (106.38 to 877.80) |
| CD4 Naive (W24) | 21 | 320.59 (124.78 to 831.21) |
| CD8 (W12) | 23 | 401.00 (125.00 to 709.00) |
| CD8 (W24) | 22 | 422.00 (129.00 to 655.00) |
| CD8 CM (W12) | 23 | 28.31 (3.34 to 155.36) |
| CD8 CM (W24) | 21 | 23.39 (3.05 to 149.12) |
| CD8 Eff (W12) | 23 | 65.77 (8.69 to 316.24) |
| CD8 Eff (W24) | 21 | 51.82 (7.49 to 383.46) |
| CD8 EM (W12) | 23 | 116.28 (4.88 to 289.91) |
| CD8 EM (W24) | 21 | 122.12 (23.65 to 308.50) |
| CD8 Naive (W12) | 23 | 66.75 (27.09 to 349.75) |
| CD8 Naive (W24) | 21 | 73.14 (17.41 to 371.31) |
| Absolute immune cell counts (cells/μL) measured by flow cytometry. | | |
| N represents the number of patients with available data. This subgroup includes placebo-randomized participants who received probiotic from Week 12 to Week 24 in the open-label phase (modified intention-to-treat complete case analysis). | | |
| CM, Central memory; Eff, Effector; Treg, Regulatory T cells. | | |

## Supplementary table S11

**Phagocytosis and oxidative burst at Baseline and Week 12**

|  | **Probiotic** | | **Placebo** | |
| --- | --- | --- | --- | --- |
|  | **N** | **Median (Min to Max)** | **N** | **Median (Min to Max)** |
| Granulocyte Phagocytosis (BSL) | 22 | 99.2 (83.9 to 99.9) | 18 | 99.3 (84.4 to 99.9) |
| Granulocyte Phagocytosis (W12) | 17 | 97.7 (76.0 to 99.9) | 14 | 97.3 (85.5 to 99.9) |
| Monocyte Phagocytosis (BSL) | 22 | 97.3 (81.5 to 99.4) | 18 | 93.3 (80.2 to 99.8) |
| Monocyte Phagocytosis (W12) | 17 | 90.3 (74.2 to 99.2) | 14 | 95.2 (78.6 to 99.1) |
| Granulocyte Oxidative Burst (BSL) | 25 | 99.5 (78.0 to 100.0) | 21 | 99.4 (96.2 to 100.0) |
| Granulocyte Oxidative Burst (W12) | 23 | 99.3 (93.7 to 100.0) | 20 | 99.0 (92.2 to 100.0) |
| Values are shown as median (range) of percentages. | | | | |
| N indicates the number of patients with available data at each timepoint according to the modified intention to treat complete case analysis.  BSL, baseline; W12, week 12 | | | | |

## Supplementary table S12

**Phagocytosis and oxidative burst at Week 12 and Week 24**

|  | **N** | **Median (Min to Max)** |
| --- | --- | --- |
| Granulocyte Phagocytosis (W12) | 12 | 97.8 (85.5 to 99.9) |
| Granulocyte Phagocytosis (W24) | 9 | 95.0 (86.1 to 99.8) |
| Monocyte Phagocytosis (W12) | 12 | 95.8 (78.6 to 99.1) |
| Monocyte Phagocytosis (W24) | 9 | 88.3 (75.6 to 97.8) |
| Granulocyte Oxidative Burst (W12) | 17 | 99.0 (92.2 to 99.8) |
| Granulocyte Oxidative Burst (W24) | 17 | 99.5 (95.9 to 100.0) |
| Values are shown as median (range) of percentages. | | |
| N represents the number of patients with available data. This includes 23 patients (minus the missing data) that were randomized to the placebo group and received the probiotic from Week 12 to Week 24 in the open-label phase, according to the modified intention to treat complete case analysis.  W12, week 12; W24, week 24 | | |

## Supplementary table S13

**Adverse events throughout 24 weeks**

| **System organ class** | **Preferred Term** | **Placebo (n=33)** | **Causality Scale** | **Probiotic (n=32)** | **Causality Scale** |
| --- | --- | --- | --- | --- | --- |
| Gastrointestinal disorders | Diarrhea | 2 (6.1%) | Unlikely | 2 (6.2%) | Possible |
|  | Constipation | 1 (3.0%) | Unlikely | 1 (3.1%) | Possible |
|  | Pancreatitis | 0 (0%) | - | 1 (3.1%)* | Unlikely |
| Hepatobiliary disorders | Liver enzyme increased | 1 (3.0%) | Unlikely | 0 (0%) | - |
| Infections and infestations | Infection | 2 (6.1%) | Unlikely | 2 (6.2%) | Unlikely |
| Skin and subcutaneous tissue disorders | Alopecia | 1 (3.0%) | Unlikely | 1 (3.1%) | Unlikely |

*All events were considered adverse events, apart from the pancreatitis case which was considered a serious adverse event due to hospitalization.

## References

1.	Apprill A, McNally S, Parsons R, Weber L. Minor revision to V4 region SSU rRNA 806R gene primer greatly increases detection of SAR11 bacterioplankton. Aquat Microb Ecol. 2015;75:129-37.

2.	Parada AE, Needham DM, Fuhrman JA. Every base matters: assessing small subunit rRNA primers for marine microbiomes with mock communities, time series and global field samples. Environ Microbiol. 2016;18(5):1403-14.

3.	Pjevac P, Hausmann B, Schwarz J, Kohl G, Herbold CW, Loy A, et al. An Economical and Flexible Dual Barcoding, Two-Step PCR Approach for Highly Multiplexed Amplicon Sequencing. Front Microbiol. 2021;12:669776.

4.	Callahan BJ, McMurdie PJ, Rosen MJ, Han AW, Johnson AJ, Holmes SP. DADA2: High-resolution sample inference from Illumina amplicon data. Nat Methods. 2016;13(7):581-3.

5.	Callahan B, Sankaran K, Fukuyama J, McMurdie P, Holmes S. Bioconductor Workflow for Microbiome Data Analysis: from raw reads to community analyses [version 2; peer review: 3 approved]. F1000Research. 2016;5(1492).

6.	McLaren, M. R., & Callahan, B. J. (2021). Silva 138.1 prokaryotic SSU taxonomic training data formatted for DADA2 [Data set]. Zenodo. <https://doi.org/10.5281/zenodo.4587955>.

7.	Quast C, Pruesse E, Yilmaz P, Gerken J, Schweer T, Yarza P, et al. The SILVA ribosomal RNA gene database project: improved data processing and web-based tools. Nucleic Acids Res. 2013;41(Database issue):D590-6.

8.	Huang R, Soneson C, Ernst F, Rue-Albrecht K, Yu G, Hicks S, et al. TreeSummarizedExperiment: a S4 class for data with hierarchical structure [version 2; peer review: 3 approved]. F1000Research. 2021;9(1246).

9.	Borman T, Ernst FGM, Shetty SA, Lahti L. mia: Microbiome analysis [Internet]. 2025 [cited 2025 Dec 4]. Available from: <https://github.com/microbiome/mia>.

10.	Oksanen J, Blanchet FG, Friendly M, Kindt R, Legendre P, McGlinn D, et al. vegan: Community Ecology Package [Internet]. R package version [cited 2025 Dec 4]. Available from: <https://CRAN.R-project.org/package=vegan>.

11.	McMurdie PJ, Holmes S. phyloseq: An R Package for Reproducible Interactive Analysis and Graphics of Microbiome Census Data. PLoS One. 2013;8(4):e61217.

12.	Lahti L, Shetty S, et al. miaverse — R/Bioconductor framework for microbiome data science [Internet]. 2025 [cited 2025 Dec 4]. Available from: <http://microbiome.github.io>.

13.	Barnett DJM, Arts ICW, Penders J. microViz: an R package for microbiome data visualization and statistics. Journal of Open Source Software. 2021 Jul 10; 6(63):3201. doi: 10.21105/joss.03201.
